# Under-Recognition of Kidney Disease in Cause-of-Death Certification: An Adjudication-Based Study With Development of an Exploratory Prediction Model

**DOI:** 10.7759/cureus.113155

**Published:** 2026-07-22

**Authors:** Anish Kumar Gupta, Narinder Pal Singh, Dinesh Khullar, Sourabh Sharma, Sonu Gupta, Gaurav Minocha, Neeru P Aggarwal

**Affiliations:** 1 Nephrology and Renal Transplant Medicine, Max Super Speciality Hospital, Saket, New Delhi, IND; 2 Medicine and Nephrology, Max Super Speciality Hospital, Saket, New Delhi, IND; 3 Nephrology, Vardhman Mahavir Medical College and Safdarjung Hospital, New Delhi, IND; 4 Medicine, Northeast Georgia Medical Center, Gainesville, USA; 5 Cardiology, Max Super Speciality Hospital, Vaishali, Ghaziabad, IND; 6 Nephrology and Renal Transplant Medicine, Max Super Speciality Hospital, Vaishali, Ghaziabad, IND

**Keywords:** acute kidney injury, cause of death, chronic kidney disease, clinical prediction, mortality

## Abstract

Background

Accurate determination of the cause of death (COD) is essential for estimating disease burden and guiding healthcare planning. The primary objective was to quantify discrepancies between death certificate documentation and adjudicated kidney disease-related deaths. The secondary objective was to develop and internally validate an exploratory prediction model for adjudicated kidney disease-related mortality.

Methods

A retrospective observational study was conducted in the medical intensive care unit (MICU) of a tertiary-care hospital in India. Medical records of 365 randomly selected deceased patients were reviewed. Kidney disease was defined to include acute kidney injury (AKI), chronic kidney disease (CKD), and acute-on-CKD, per KDIGO criteria. Candidate predictors were screened using Information Value (IV) and Variance Inflation Factor (VIF) analyses, followed by multivariable logistic regression. The model was developed in a 75% training cohort and validated in the remaining 25%. Performance was assessed using accuracy, precision, recall, F1-score, and area under the receiver operating characteristic curve (AUROC). Odds ratios (ORs) with 95% confidence intervals (CIs) were reported, and model coefficients were used to develop a risk calculator for predicting kidney disease-related death.

Results

Kidney disease was identified as contributing to death in 67.4% of cases following detailed chart review, compared with 45.2% documented on death certificates, indicating substantial underreporting. The present model identified oliguria (OR 20.48, 95% CI 8.02-52.31; p < 0.001) and hemodialysis (OR 13.46, 95% CI 2.65-68.47; p = 0.002) as the strongest predictors of kidney disease-related death, whereas hypotension, total protein, and cancer demonstrated borderline associations. The model demonstrated good predictive performance, achieving accuracies of 84.6% and 72.8% in the training and validation datasets, respectively. Discriminatory ability was excellent, with AUROC values of 0.905 in the training cohort and 0.856 in the validation cohort, indicating good model stability and generalizability.

Conclusions

COD attributable to kidney disease appears to be substantially underreported in death certification. A simple clinical prediction model demonstrated promising discriminatory performance in this exploratory analysis and may support more accurate attribution of kidney disease-related mortality. External validation in diverse healthcare settings is required before wider implementation.

## Introduction

Accurate estimation of disease burden is fundamental for developing effective public health policies and allocating healthcare resources appropriately. Cause of death (COD) statistics constitute a key component of this process, providing essential information for health planning and priority setting. Kidney disease represents a significant global public health concern and is associated with substantial morbidity and mortality [1]. According to the Global Burden of Disease (GBD) 2023 report, kidney disease ranked as the ninth leading COD worldwide, accounting for approximately 1.48 million deaths annually [2]. However, determining mortality attributable to kidney disease remains challenging in many developing countries due to incomplete vital registration systems and limited accuracy of death certification.

In India, inaccuracies in COD reporting may arise from inadequate certification practices, limited training in death certification, and the tendency to assign deaths to ill-defined or nonspecific categories. Though both AKI and CKD are recognized contributors to premature mortality, various mortality surveillance systems fail to capture the full contribution. This is because death certificates endorse immediate terminal events only, such as sepsis, respiratory failure, or cardiac arrest. Hence, there is underestimation of kidney disease burden and inadequate resource allocation. Data evaluating this discrepancy in low- and middle-income countries remains scarce, including in India.

The primary objective of this study was to quantify the discrepancy between routine death certificate documentation and adjudicated kidney disease-related deaths using an independent expert review process. The secondary objective was to develop and internally validate an exploratory prediction model for identifying adjudicated kidney disease-related deaths.

## Materials and methods

A retrospective observational study was conducted at a tertiary-care hospital that caters to the urban and rural populations in and around the National Capital Region of Delhi, India. The study center had a wide variety of high-acuity cases due to its large catchment area and easy accessibility by both health-insured and uninsured patients. The study was approved by the institutional ethics committee.

Study population

The study was conducted in the medical intensive care units (MICUs) of a tertiary-care referral hospital. The MICUs provide care for critically ill adult patients with a wide range of medical conditions, including sepsis, septic shock, acute kidney injury (AKI), chronic kidney disease (CKD), cardiovascular diseases, respiratory illnesses, malignancies, and multi-organ dysfunction syndrome. Only deceased patients admitted under medical specialties and managed in the MICUs were eligible for inclusion. Patients admitted to surgical intensive care units were not included in the study. Cases with incomplete clinical documentation, missing essential information required for COD adjudication, duplicate records, or uncertain final diagnoses were excluded from the analysis. CKD and AKI were defined according to KDIGO guidelines [3,4].

Sampling

A sampling frame consisting of all deceased patients admitted to the MICU between January 2021 and December 2023 was generated from the hospital mortality database. Each eligible case was assigned a unique identification number. Simple random sampling was performed using a computer-generated random number sequence to select patient records for inclusion. This approach ensured that every eligible deceased patient had an equal probability of selection and minimized selection bias.

The required sample size was estimated using Cochran's formula, assuming a prevalence of 50%, a 95% confidence level (CI), a margin of error of 5.4%, and a 10% attrition rate, yielding a target sample size of 365 patients.

Data collection

Data were collected through retrospective review of hospital medical records, death certificates, laboratory reports, and other relevant source documents using a structured data abstraction form. The major underlying COD was considered using a restricted list as listed in the 10th version of the International Classification of Diseases (ICD-10). The diagnoses selected for inclusion in the list were derived from the death certificate/death summary sheet. We did not exclude certificates that mentioned multiple causes of death for a specific patient.

Because no universally accepted gold standard exists for determining the COD attributable to kidney disease, a priori adjudication criteria were developed based on KDIGO definitions of AKI and CKD, published literature, and expert consensus among nephrologists and intensivists. Death was classified as attributable to kidney disease when kidney disease was identified as the primary underlying COD or as a major contributor to the chain of clinical events leading to death. Specifically, kidney disease was considered contributory if one or more of the following conditions were present: (i) AKI, CKD, acute-on-CKD, glomerulonephritis, or other significant renal pathology was the principal diagnosis leading to death; (ii) renal complications such as severe uremia, refractory hyperkalemia, metabolic acidosis, or fluid overload directly contributed to death; (iii) dialysis dependence or dialysis-related complications played a major role in the terminal illness; or (iv) kidney disease substantially worsened another fatal condition and contributed to the sequence of events culminating in death.

COD Adjudication

The COD for each selected case was independently reviewed by two senior physicians with expertise in nephrology and internal medicine. Reviewers examined the complete medical record, including admission notes, progress notes, laboratory investigations, imaging findings, dialysis records, discharge summaries, and death certificates. During the initial review, adjudicators were blinded to each other's assessments and independently classified deaths as kidney disease-related or non-kidney disease-related according to predefined study criteria. Cases with discordant classifications were subsequently discussed in a consensus meeting. If disagreement persisted, the final classification was determined through review by the institutional mortality review committee. Inter-rater agreement between the two reviewers was assessed using Cohen's kappa statistic. Cohen's κ was calculated based on the initial independent assessments of the two reviewers before consensus adjudication.

Risk model development and validation 

Statistical Analysis

Statistical analyses were performed using Python-based statistical packages. The outcome variable was COD attributable to kidney disease, classified as a binary outcome (Yes = 1 and No = 0) based on adjudicated source-document review. Candidate predictor variables included demographic characteristics, comorbidities, clinical features, laboratory parameters, and established risk factors for AKI and CKD. Variable selection was guided by clinical relevance together with Information Value (IV) analysis and multicollinearity assessment. Initially, IV analysis was performed to assess the predictive strength of candidate variables. Variables demonstrating acceptable discriminatory ability (IV > 0.10) were considered for multivariable model development. To minimize multicollinearity, Variance Inflation Factor (VIF) analysis was performed. Variables with VIF ≥ 5 were considered highly collinear and were excluded from the final model (Appendix 1). The remaining variables were entered into a multivariable logistic regression model using maximum likelihood estimation to identify independent predictors of kidney disease-related death.

For model development and internal validation, the dataset was randomly divided using a computer-generated random allocation procedure into a training cohort (75%) and an independent validation cohort (25%). The prediction model was developed using the training dataset and subsequently evaluated in the validation dataset. The significance of individual predictors was assessed using the Wald χ² test (df = 1 for each predictor), while the overall model fit was evaluated using the likelihood ratio test (df = 10). Model performance was assessed by calculating accuracy, precision, recall (sensitivity), F1-score, and the area under the receiver operating characteristic curve (AUROC). Odds ratios (ORs) with corresponding 95% CIs were reported for significant predictors. A two-sided p-value < 0.05 was considered statistically significant. No missing data were observed for the variables included in model development.

Model Development: Electronic Risk Calculator

The final multivariable logistic regression model incorporated independent predictors identified during model development. Regression coefficients derived from the final model were used to construct the prediction equation and develop a user-friendly electronic risk calculator for estimating the probability that kidney disease was attributable to the COD. The methodology adopted for prediction modelling and manuscript preparation was in accordance with the TRIPOD and STROBE statements [5,6].

## Results

Characteristics of the study population

General characteristics (demographic/clinical) of the study cohort are demonstrated in Table 1. Of the 365 deceased cases, 60.5% were men, with a mean age of 65 years (SD 14.17). Overall, 70.6% of deaths occurred among individuals aged 60 years and above, while 5.7% occurred among those aged 40 years or younger. A history of diabetes, hypertension, cardiovascular disease, malignancy, and CKD was found in 45%, 45.4%, 30%, 24.9%, and 25.7% of cases, respectively. Most cases were admitted to the medical ICU (68.7%), followed by oncology (17.7%) and the internal medicine unit (15.3%). COD was selected from the ICD-10 (Table 1). More than two-thirds of all deaths were attributable to infectious aetiologies, such as sepsis, septic shock, and multi-organ dysfunction. Following this, diseases of the genitourinary system accounted for 53%. Diseases of the respiratory system accounted for one in every three deaths. Following these were endocrine, neoplastic, and digestive system diseases, accounting for 20%, 25.4%, and 24%, respectively.

**Table 1:** Cause of death reported in death certificate/death summary sheet (list derived from ICD-10) * Percentages sum to >100% because multiple causes of death were recorded per patient on death certificates/summary sheets. CKD: chronic kidney disease; ICD: International Statistical Classification of Diseases

Cause of death	n=365
Male n(%)	221 (60.5)
Age, years, mean (SD)	65 (14.17)
<20, n(%)	2 (0.5)
20-40, n(%)	19 (5.2)
41-60, n(%)	93 (25.5)
61-80, n(%)	208 (57)
>80, n(%)	43 (11.8)
Diabetes, n (%)	164 (45)
Hypertension, n (%)	166 (45.4)
Cardiovascular disease, n (%)	110 (30)
Malignancy, n (%)	91 (24.9)
CKD, n (%)	94 (25.7)
Cause of death (ICD-10 classification), n (%)*	
Certain infectious and parasitic diseases (A00-B99)	241 (66)
Neoplasms (C00-D48)	93 (25.5)
Diseases of blood-forming organs and immune mechanism disorders (D50-D89)	15 (4.1)
Endocrine, nutritional and metabolic diseases (E00-E90)	75 (20.5)
Diseases of the nervous system (G00-G99)	25 (7)
Diseases of the circulatory system (I00-I99)	138 (37.8)
Diseases of the respiratory system (J00-J99)	106 (29)
Diseases of the digestive system (K00-K93)	88 (24)
Diseases of the genitourinary system-kidney disease/UTI (N00-N99)	197 (54)
Pregnancy, childbirth and the puerperium (O00-O99)	1 (0.2)
Symptoms and abnormal clinical/laboratory findings, not elsewhere classified (R00-R99)	192 (52.6)
Injury, poisoning, and certain other consequences of external causes (S00-T98)	11 (3)
Right lower limb cellulitis	1 (0.2)

Kidney disease was identified as contributing to death in 67.4% of cases following detailed chart review and adjudication, whereas only 45.2% of death certificates documented kidney disease as a COD (Table 2). This finding suggests substantial under-recognition of kidney disease in routine mortality reporting. Agreement between the two independent reviewers was 87.3%, with a Cohen's κ of 0.78, indicating substantial inter-rater reliability in the classification of kidney disease-related deaths.

**Table 2:** Agreement between death certificate and adjudicated cause of death

Classification	Death certificate	Adjudication
Kidney disease-related death	165 (45.2%)	246 (67.4%)
Not kidney disease related	200 (54.8%)	119 (32.6%)
Absolute difference	-	+22.2%
Relative increase in recognition	-	+49%

Of the 365 deaths reviewed, 56 cases (15.4%) required consensus adjudication owing to disagreement between the two primary reviewers.

Development of a risk prediction model

A total of 365 deceased patients were included in the analysis, of whom 246 (67.4%) were classified as having death attributable to kidney disease and 119 (32.6%) as having death due to non-kidney-related causes. IV analysis was performed to identify candidate predictors of kidney disease-related mortality. Oliguria (IV = 1.84) and hemodialysis (IV = 1.47) demonstrated the strongest discriminatory power, followed by age, hemoglobin, CKD, systolic blood pressure, total protein, hypotension, circulatory shock, serum sodium, albumin, dehydration/volume depletion, sepsis, diastolic blood pressure, cancer, and trauma.

Multicollinearity was assessed using VIFs. Variables with excessive collinearity were sequentially excluded until all retained variables demonstrated acceptable VIF values (<5). Variables exhibiting substantial collinearity, including serum sodium, systolic blood pressure, age, hemoglobin, albumin, and circulatory shock, were sequentially excluded. The final multivariable model retained ten predictors: oliguria, hemodialysis, CKD, total protein, hypotension, dehydration/volume depletion, sepsis, diastolic blood pressure, cancer, and trauma. All retained variables demonstrated acceptable VIF values (<5).

The final multivariable logistic regression model showed a significant improvement over the null model (likelihood ratio test, df = 10, p < 0.001). The significance of individual predictors was assessed using Wald χ² statistics (df = 1 for each predictor). Among the retained predictors, oliguria and hemodialysis emerged as the strongest independent determinants of kidney disease-related mortality. Patients with oliguria had a markedly increased likelihood of kidney disease-related death compared with those without oliguria (OR 20.48, 95% CI 8.02-52.31; p < 0.001). Similarly, patients receiving hemodialysis had significantly higher odds of kidney disease-related mortality (OR 13.46, 95% CI 2.65-68.47; p = 0.002).

Several additional variables demonstrated borderline significance at the 90% CI. Hypotension was associated with an increased likelihood of kidney disease-related mortality (OR 2.25, 95% CI 0.99-5.09; p = 0.052), whereas higher total protein levels showed a trend toward a protective effect (OR 0.90, 95% CI 0.79-1.01; p = 0.071). Cancer was inversely associated with kidney disease-related mortality (OR 0.47, 95% CI 0.21-1.08; p = 0.075), potentially reflecting competing non-renal causes of death. However, these associations did not achieve statistical significance at the conventional 95% confidence level and should therefore be interpreted with caution. In addition, CKD, sepsis, dehydration/volume depletion, diastolic blood pressure, and trauma demonstrated acceptable discriminatory ability based on IV analysis (Appendix 1); however, none remained significantly associated with kidney disease-related mortality in the multivariable model at the 10% level of significance (all p > 0.10) (Table 3).

**Table 3:** Final multivariable logistic regression model and relative contribution of predictors to kidney disease-related mortality attribution † Hemodialysis was retained in the model as a marker of severe renal dysfunction and should be interpreted as a mediating indicator rather than an independent causal predictor. Contribution (%) was calculated as the proportion of each predictor's Wald χ² statistic relative to the sum of Wald χ² statistics for all predictors retained in the final model: $\text{Contribution (\%)} = \left[\frac{\text{Wald } \chi^2 \text{ of predictor}}{\sum(\text{Wald } \chi^2 \text{ of all predictors})}\right] \times 100$. Variables demonstrating acceptable discriminatory ability during IV analysis and contributing to overall predictive performance were retained despite not achieving conventional statistical significance. OR: odds ratio; CI: confidence interval; CKD: chronic kidney disease

Predictor	β Coefficient	OR	95% CI	p-value	Wald χ²	Contribution (%)
Oliguria	3.0196	20.48	8.02-52.31	<0.001	39.85	61.9
Hemodialysis†	2.6000	13.46	2.65-68.47	0.002	9.82	15.3
Hypotension	0.8106	2.25	0.99-5.09	0.052	4.75	5.9
Total protein (g/dL)	-0.1107	0.90	0.79-1.01	0.071	3.26	5.1
Cancer	-0.7484	0.47	0.21-1.08	0.075	3.17	4.9
Trauma	-1.9625	0.14	0.01-1.62	0.112	2.50	3.9
Diastolic blood pressure (mmHg)	-0.0062	0.99	0.981-1.006	0.328	0.96	1.5
Sepsis	0.2782	1.32	0.61-2.88	0.484	0.49	0.8
History of CKD	0.4092	1.51	0.45-5.02	0.505	0.45	0.7
Dehydration/volume depletion	0.2757	1.32	0.21-8.34	0.768	0.09	0.1

The prediction model can be described using the following mathematical equation: $\mathrm{Logit}(p) = -1.7222 + 3.0196(\mathrm{Oliguria}) + 2.6000(\mathrm{Hemodialysis}) + 0.4092(\text{history of chronic kidney disease}) - 0.1107(\text{Total protein}) + 0.8106(\mathrm{Hypotension}) + 0.2757(\text{Dehydration/volume depletion}) + 0.2782(\mathrm{Sepsis}) - 0.7484(\mathrm{Cancer}) - 1.9625(\mathrm{Trauma}) - 0.0062(\text{Diastolic blood pressure})$

The corresponding probability of kidney disease-related mortality was calculated as: $p = \frac{1}{1 + e^{-\mathrm{Logit}(p)}}$, where p = predicted probability that the death was attributable to kidney disease and e = Euler's constant (approximately 2.71828). Binary variables were coded as follows: Present = 1; Absent = 0. Continuous variables were entered as measured values: total protein (g/dL) and diastolic blood pressure (mmHg).

The model demonstrated good predictive performance. In the training dataset, the model achieved an accuracy of 84.6%, precision of 85.1%, recall of 93.5%, and F1-score of 89.1%. In the independent test dataset, performance remained satisfactory, with an accuracy of 72.8%, precision of 76.1%, recall of 87.1%, and F1-score of 81.2%, indicating good stability and generalizability.

**Figure 1:**
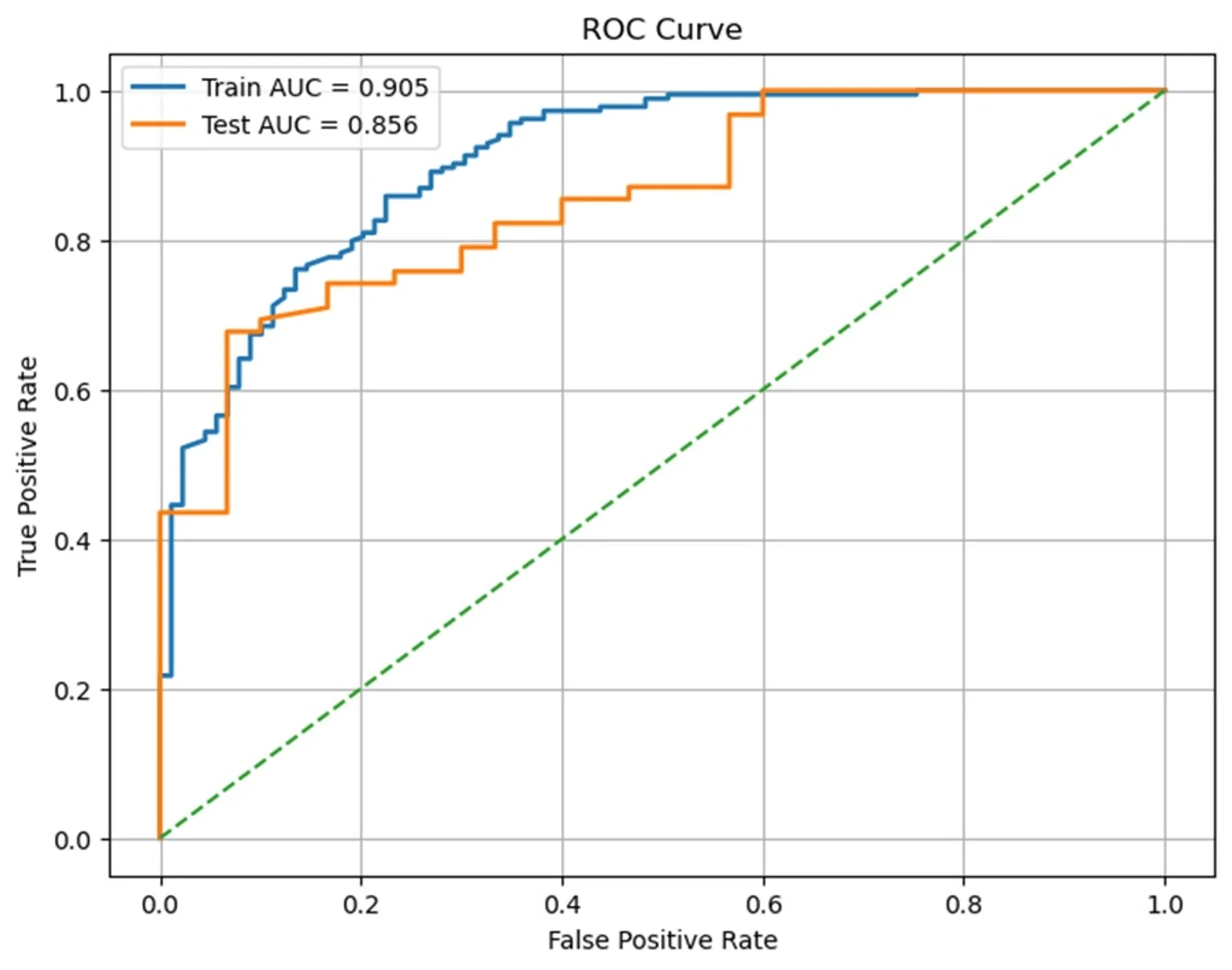
ROC curves of the final kidney disease mortality attribution model in the training and validation cohorts AUC: area under the curve; ROC: receiver operating characteristic

ROC analysis further confirmed the discriminatory ability of the model. The AUROC was 0.905 in the training cohort and 0.856 in the validation cohort, indicating excellent discrimination and good external performance within the study population (Figure 1).

## Discussion

Kidney disease is a major public health challenge, even though the magnitude of the problem is often under-recognized [7]. Recent data from India, which contributes around one-fifth of global deaths, shows kidney disease as the burgeoning cause of premature adult mortality [8]. There is a reported high prevalence of low eGFR in India, ranging from 3.8% to 4.2% (using the Modification of Diet in Renal Disease (MDRD) equation), similar to that in many other tropical nations [9,10]. In view of this temporal trend, large population size, and rapid demographic transitions, renal disease estimates from India are vital for understanding global health statistics. Notwithstanding the importance of delineating the epidemiology of renal disease in India, existing data are sporadic and highlight the inadequacies of using data from COD reporting systems [11,12]. The GBD-2016 study in India reported inequalities in disease burden across the nation [13]. The GBD-2015 report observed evidence of gaps in empirical data obtained through COD estimates [2]. Specifically, in the context of kidney disease, it has been seen that the documentation of underlying kidney disease-related deaths is erroneous and misclassified [2]. Compounding this problem is the fact that, given non-specific symptoms and the presence of multiple co-morbidities, verbal autopsy may not be accurate in ascertaining kidney disease-related deaths [14]. In view of this, it is necessary to evolve a process or prediction tool to accurately document kidney disease as the COD that, if validated, could be widely used by medical practitioners during death certification.

In the present study, kidney disease was identified as contributing to death in 67.4% of cases following detailed chart review and adjudication, whereas only 45.2% of death certificates documented kidney disease as a COD. This finding suggests substantial under-recognition of kidney disease in routine mortality reporting. Several factors may contribute to underreporting. Immediate terminal events, such as sepsis, septic shock, respiratory failure, or multi-organ dysfunction syndrome, are often documented as causes of death, whereas underlying renal disease may be omitted. Additionally, inadequate training in death certification and the complexity of critically ill patients may contribute to incomplete attribution of kidney disease.

The discrepancy observed in our study also reflects the tendency of physicians to record immediate physiological causes of death. The underlying chronic disease processes are often missed. Similar patterns have been reported for other chronic diseases, viz. diabetes, dementia, and chronic liver disease.

Under-recognition of kidney disease in mortality statistics also has important policy implications. Mortality estimates inform national health priorities and funding allocations. They also inform workforce planning, dialysis infrastructure development, and preventive CKD screening programmes. Systematic underreporting may lead to underinvestment in kidney care services.

In view of the growing importance of kidney disease, tools for the accurate determination of mortality due to kidney disease are much needed. In the present study, we attempted to construct an exploratory prediction model by utilising the deceased patients’ demographic details, acute and chronic comorbidities, and risk factors associated with kidney injury, and demonstrated that the model shows potential to help estimate the probability of death attributable to kidney disease. The key findings of the study and their potential implications are summarized in Table 4.

**Table 4:** Interpretation of key findings and potential implications *Post-hoc summary table presented for interpretive clarity; not a pre-specified study outcome. AUROC: area under the receiver operating characteristic curve; AKI: acute kidney injury

Key finding*	Possible explanation	Clinical implications	Public health implications
67.4% deaths adjudicated as kidney-related	Detailed chart review identified kidney contribution not captured in routine certification	Increased clinician awareness needed	True burden of kidney disease may be underestimated
Only 45.2% death certificates mentioned kidney disease	Focus on terminal events rather than underlying disease	Need for physician training in death certification	Mortality statistics may be inaccurate
Oliguria strongest predictor	Marker of severe AKI and organ dysfunction	Should trigger consideration of kidney-related death	Useful variable in mortality surveillance
Hemodialysis strongly associated with kidney-related death	Reflects advanced kidney dysfunction	Facilitates risk stratification	May identify high-risk populations
AUROC 0.905	Good model discrimination	Potential support tool for mortality review	Requires external validation before implementation
Substantial reviewer agreement (κ = 0.78)	Structured adjudication process	Supports reproducibility	Could be incorporated into mortality audits

The present model identified oliguria and hemodialysis as the strongest predictors, while hypotension, total protein, and cancer demonstrated borderline associations. Although CKD, sepsis, dehydration/volume depletion, diastolic blood pressure, and trauma demonstrated acceptable discriminatory ability in IV analysis, their associations did not attain statistical significance in the multivariable model. Nevertheless, these variables were retained in the final probability equation because of their contribution to the overall predictive performance of the model.

The final prediction model was designed to maximize discrimination of kidney disease-related mortality rather than to establish causal relationships between predictors and outcome. Consequently, variables such as hemodialysis were retained because of their strong predictive contribution, despite representing markers of disease severity. Oliguria is a recognized marker of severe renal dysfunction and has consistently been associated with adverse outcomes and increased mortality in AKI and advanced kidney disease populations. The observed trend toward increased mortality among patients with hypotension may be explained by impaired renal perfusion and worsening kidney injury, mechanisms that are well-recognized contributors to adverse renal outcomes. The inverse association observed with cancer likely reflects competing causes of death, whereby patients with malignancy frequently succumb to cancer-related complications rather than kidney disease itself.

The risk model was developed using readily available clinical variables, including established susceptibility and exposure-related risk factors for kidney disease recognised by the KDIGO guidelines [3,4]. These risk factors have also been used to develop risk models to predict the occurrence of AKI in various clinical contexts, such as AKI in the ICU in paediatric or adult populations or contrast-induced nephropathy [15-22]. Similar to our study, but in a different context, a prediction model for AKI in ICU settings integrated acute sequelae with chronic comorbidities at the time of ICU admission to identify individuals at high risk for AKI [22]. Our study is unique in that it used the same methodology to help predict adjudicated kidney disease-related death. Our model had an AUROC of 0.905, indicating strong discrimination within this internal cohort.

The model is intended as a proof-of-concept tool to support the identification of deaths potentially attributable to kidney disease during mortality review and death certification. It is not intended to replace clinical judgment. Although the model demonstrated excellent internal discrimination, external validation has not yet been performed. Therefore, the model should be considered exploratory and hypothesis-generating.

This study has a few limitations. First, it was a retrospective single-center study conducted in a tertiary-care hospital, which may limit the generalizability of the findings to other healthcare settings. Second, because no universally accepted gold standard exists for attributing death to kidney disease, the adjudicated classification used in this study represents a pragmatic reference standard based on expert review and predefined criteria. Although measures were taken to minimize bias, some degree of subjectivity and misclassification cannot be completely excluded. Third, the relatively modest sample size and inclusion of only deceased patients admitted to medical ICUs may have introduced selection bias and limited the broader applicability of the model. The sample size was calculated a priori, assuming a conservative 50% prevalence, as no prior local data were available. Since this assumption maximizes the required sample size for a given margin of error, the observed prevalence of 67.4% yields a narrower post-hoc margin of error (~4.8%) than the ~5.4% originally specified, confirming adequate precision. Fourth, although internal validation demonstrated excellent discrimination, external validation was not performed; therefore, the model's performance in other populations remains uncertain. Fifth, selection of the model with the highest AUROC during cross-validation may have resulted in optimistic estimates of predictive performance. Sixth, the possibility of incomplete documentation and residual confounding from unmeasured clinical factors cannot be excluded. Finally, the possibility of incorporation bias cannot be excluded, as certain clinical variables, particularly oliguria and hemodialysis, may have contributed to both COD adjudication and model development. Consequently, the model should be interpreted as an exploratory mortality-attribution tool rather than a causal model.

## Conclusions

Kidney disease as a COD appears to be substantially underreported on routine death certificates. Using detailed hospital records and expert adjudication, we developed a simple clinical risk prediction model based on readily available patient characteristics and kidney disease risk factors that demonstrated promising discriminatory performance in this exploratory, internally validated analysis. If externally validated, this model may serve as a practical tool to improve COD certification, enhance the accuracy of mortality statistics, and support more informed public health planning and resource allocation for kidney disease.

## Appendices

Appendix 1

**Table 5:** Information value (IV) and variance inflation factor (VIF) analysis of candidate predictors for development of the kidney disease mortality attribution model *Information Value (IV) interpretation: <0.02 = not predictive; 0.02-0.10 = weak; 0.10-0.30 = medium; 0.30-0.50 = strong; >0.50 = very strong. Variables with excessive multicollinearity (VIF >5) were sequentially excluded from the multivariable logistic regression model. The final model retained predictors with acceptable VIF values (<5) while preserving discriminatory performance identified through IV analysis.

Variable	Information value (IV)	Discriminatory strength*	VIF	Status in final model
Oliguria	1.84	Strong	1.15	Retained
Hemodialysis	1.47	Strong	1.32	Retained
Age	0.62	Strong	13.78	Excluded (High VIF)
Hemoglobin	0.54	Strong	8.27	Excluded (High VIF)
CKD	0.46	Medium	1.87	Retained
Total Protein	0.36	Medium	1.41	Retained
Hypotension	0.31	Medium	2.24	Retained
Circulatory Shock	0.28	Medium	6.47	Excluded (High VIF)
Serum Sodium	0.26	Medium	30.86	Excluded (High VIF)
Albumin	0.23	Medium	6.68	Excluded (High VIF)
Diastolic Blood Pressure	0.142	Medium	2.52	Retained
Dehydration/Volume Depletion	0.19	Weak	1.22	Retained
Sepsis	0.17	Weak	1.35	Retained
Cancer	0.13	Weak	1.18	Retained
Trauma	0.11	Weak	1.09	Retained
